# Editorial: Hybrid brain-robot interfaces for enhancing mobility

**DOI:** 10.3389/fnbot.2023.1264045

**Published:** 2023-08-15

**Authors:** Stefano Tortora, Fiorenzo Artoni, Silvestro Micera, Luca Tonin, Solaiman Shokur

**Affiliations:** ^1^Department of Information Engineering, University of Padova, Padova, Italy; ^2^Padova Neuroscience Center, University of Padova, Padova, Italy; ^3^Functional Brain Mapping Laboratory, Department of Basic Neuroscience, Faculty of Medicine, University of Geneva, Geneva, Switzerland; ^4^Translational Neural Engineering Lab, Institute Neuro X, École Polytechnique Fédérale de Lausanne, Geneva, Switzerland; ^5^Department of Excellence in Robotics and AI, Institute of BioRobotics Interdisciplinary Health Center, Scuola Superiore Sant'Anna, Pisa, Italy

**Keywords:** brain-robot interface (BRI), electromyography (EMG), electroencephalography (EEG), sense of agency (SoA), neuroprosthesis

Recent years have seen a strong research effort in the field of neurotechnologies and neuroprosthetics—technologies that interact with the user's nervous system to restore lost or impaired neurological functions. For example, bionic limbs have allowed people with amputation to recover significant levels of dexterity (Mendez et al., 2021), and the stimulation of the spinal cord has suggested the possibility to restore locomotion in people with paralysis (Wagner et al., 2018) (see Losanno et al., 2023 for a review). However, with the advancement of these assistive devices, there is a need for the decoding of the motor intention to allow users to control their devices in an *intuitive* and *robust* manner.

In this direction, brain-machine interfaces (BMIs) have been largely proposed and exploited to recognize user's intentions through the decoding of cortical neural patterns and translate them into actions for a variety of assistive devices, such as wheelchairs, telepresence robots, exoskeletons, and robotic arms (Tonin and Millán, 2021). Despite the substantial improvements achieved by recent BMI systems, neurorobots driven by the sole brain activity are not fully acceptable in both clinical and home-care settings. Indeed, BMIs based on non-invasive recording, e.g., electroencephalography (EEG), are generally characterized by low accuracy and low informative content due to the intrinsic unreliability and non-stationarities of the driving signals. On the other hand, invasive BMIs provide clearer brain signals, potentially enhancing the decoding of users' intentions, but come with higher costs and risks due to the required surgical intervention. All these limitations lead to a higher workload required to use these neurotechnologies in everyday life, slowing down their acceptance and their translational impact.

To overcome these limitations, a recent approach proposes to integrate different human-machine interfacing modules to either improve the accuracy of the motor decoding or increase the number of degrees-of-freedom (DoFs) for controlling the robotic device (Shokur et al., 2021), and the development of novel software frameworks for neurorobotics (Tonin et al., 2022). This approach led to the development of hybrid brain-machine interface (h-BMI) solutions combining a BMI system with at least one additional (neuro)physiological interface (Hong and Khan, 2017), such as electromyography (Tortora et al., 2020) or electrooculography (Huang et al., 2019). In this sense, it would be desirable to have a variety of motor decoding modules that could be integrated and merged in order to personalize the neurotechnology to the specific user's needs, possibilities, and preferences. The current Research Topic gives an overview of such modules.

Connan et al. investigated the use of a tactile myography interface to decode the motor intent from the measurement of the volumetric variation of the muscles during contraction. This technique is an interesting alternative to more standard electromyography decoding. In the proposed experimental scenario, the participants were first asked to perform single hand and wrist actions (e.g., rest, power, flexion, extension, pronation, and supination). The recorded data were used to train the decoding algorithm. They demonstrated that the proposed interface is able to decode both single and combined actions and can be proficiently used to control two DoFs in an online goal-reaching task.

When residual movements of the upper and lower limbs are lacking due to a severe motor impairment, the user may benefit from alternative modalities relying on motor functions that are still available. Mohammadi et al. proposed a control interface for an assistive upper-limb exoskeleton with five DoFs based on an intraoral tongue-computer interface (ITCI) for individuals with tetraplegia. They showed not only that the proposed ITCI can be used to successfully control the exoskeleton in an activity of daily living without visual feedback, but also that it can achieve performance comparable to a standard joystick.

An alternative to using already available motor functions is to learn a new function for controlling a neuroprosthesis. Pinheiro et al. demonstrated the feasibility of using auricular muscles to control a visual cursor. The proposed method exploits the electromyographic recording of auricular muscle contraction to allow a self-paced continuous modulation of the cursor's velocity and position in a 2D space. In their experiment, they show that naive users can achieve successful control of the system with short training time. The main advantage of this approach is that auricular muscles are vestigial muscles that are not normally used, and they could thus represent an ideal solution for restoring a missing motor function without hindering other residual ones.

The final goal is to go beyond the current human-machine interfacing and achieve a more natural human-robot integration. Cornelio et al. suggest that the sense of agency (SoA)—the experience of feeling in control when voluntarily interacting with the assistive technology—represents a key aspect to obtain such an integration. To shed light on the concept of *agency*, their review paper proposes a categorization of the key elements that compose the SoA, describing how agency arises from each category of human-robot interaction. The aim is to provide the research community with guidelines to better understand and enhance the SoA in their systems, as well as discuss the opportunities and challenges to obtain a tight integration between humans and technology.

Despite the importance of SoA in the development of innovative neurotechnology, quantitative approaches to measure the rise of agency in human-robot interaction are still scarce, and qualitative questionnaire-based evaluations are mostly used. Marchesotti et al. address this issue by investigating neural correlates of visuomotor integration in human-machine interaction. In their experiment, they employed a bimanual robotic interface. During the interaction, the participants were provided with experimentally manipulated visual feedback through a virtual reality system while somatosensory evoked responses were measured through EEG recording. The group showed that the activity of the right posterior parietal cortex is responsive to the incongruency in the human-robot interaction and is correlated with a reduced sense of agency experienced by the subjects.

In conclusion, the current Research Topic has presented several new directions for motor decoding modules for assistive devices. These modules could add to the already large catalog of motor decoding strategies, such as the use of joysticks, eye tracking, sip-and-puff, or BMIs. Hybrid solutions integrating several of these modules can be a pragmatic solution to propose personalized solutions and foster the translation of brain-robot interfaces into clinical applications for enhancing mobility.

## Author contributions

ST: Conceptualization, Writing—original draft. FA: Writing—review and editing. SM: Writing—review and editing. LT: Writing—review and editing. SS: Supervision, Writing—original draft.
